# Racial and Ethnic Disparities in Teleworking Due to the COVID-19 Pandemic in the United States: A Mediation Analysis

**DOI:** 10.3390/ijerph19084680

**Published:** 2022-04-13

**Authors:** Abay Asfaw

**Affiliations:** Centers for Disease Control and Prevention (CDC), National Institute for Occupational Safety and Health (NIOSH), Washington, DC 20201, USA; aasfaw@cdc.gov

**Keywords:** teleworking, racial and ethnic disparities, occupation, college education, mediation analysis, current population survey, COVID-19

## Abstract

A growing literature has pointed out disparities in teleworking among different racial and ethnic (hereafter racial) workers. This study estimated racial disparities in teleworking due to the COVID-19 pandemic and the extent to which these disparities were mediated by four-year college education and occupation in the United States. The data source for this study was the Current Population Survey, May 2020 through July 2021. The results showed that in the reduced model, the odds for Black and Hispanic workers to telework were 35% and 55% lower, respectively, and for Asian workers 44% higher than for White workers, controlling for covariates. When four-year college education and occupation were included as mediator variables in the model, the odds for Black and Hispanic workers to telework were reduced to 7% and 16%, respectively. Overall, disparities in four-year college education and occupation explained 83% and 78% of the variation in the odds of teleworking for Black and Hispanic workers, respectively. Between the mediators, occupation explained more than 60% of the total effect. The results of this study could not rule out the possibility of racial discrimination in teleworking. Ultimately, reducing racial disparities in four-year college education and in different occupations might be a long-term solution for reducing racial disparities in teleworking.

## 1. Introduction

Teleworking, also known as telecommuting or virtual working, is an alternative type of work arrangement that allows workers to perform some or all their work from home during paid work hours with no personal contact with co-workers or customers using information technology [1,2,3,4]. Advancement in information technology, personal computers, virtual platforms, and organizational structure have accelerated the expansion of teleworking [1,5,6]. Using different models and perspectives, researchers have examined the potential benefits, pitfalls, and trends of teleworking in different parts of the world [7,8,9,10,11] and personal, motivational, and situational factors that affect teleworking [12,13,14,15,16,17]. There are also comprehensive literature reviews on the experience, challenges, directions, health effects, and other implications of teleworking [18,19,20,21,22,23]. The literature shows several benefits of teleworking for both employers and employees. Some of the potential benefits of teleworking to employers include improved recruitment and retention of different types of workers [24,25,26,27], increased productivity [24,26,28,29], reduced absenteeism [30], and reduced expenses related to office space and other operating expenses [2,31]. The benefits to employees include improved work/family balance [24,28,32,33,34], increased morale and job satisfaction [28,35], and reduced commute time [36]. Some challenges of teleworking are also documented in the literature including difficulty of separating work and family life [37], lack of career development and promotions due to out-of-sight, out-of-mind syndrome [38,39,40,41,42], and professional isolation [43,44]. Overall, if properly implemented, teleworking could help improving the performance of both employees and organizations [18,27].

The COVID-19 pandemic has revealed the importance of teleworking in protecting eligible workers’ safety and reducing job losses and use of unemployment benefits [6,45,46]. The Centers for Disease Control and Prevention (CDC) reported that teleworking could significantly reduce the probability of testing positive for COVID-19 [47]. Both for performance and safety of workers, government agencies and companies have adopted different policies to maximize teleworking options for their workers. Blzunegui-Eraso and Erro-Garcés [6] showed that during the early months of the pandemic several companies in the United States developed and implemented teleworking polices to protect workers’ health and declining productivity. In May 2020, 37.6% of workers were teleworking due to the pandemic [48]. Across Europe, the percentage of workers who teleworked at least occasionally increased from 11% to 48% during the pandemic [49]. A growing literature has pointed out that teleworking has been lower among non-Hispanic Blacks and Hispanics than non-Hispanic Whites and non-Hispanic Asians in the United States [45,50,51]. Even before the pandemic, Hispanic and Black workers were 50% less likely to telework regularly compared to non-Hispanic White workers [45]. In May 2020, the percentage of employed Hispanic and non-Hispanic Black workers teleworking due to COVID-19 were 24.3 and 30.9, respectively, compared to 40.6% of non-Hispanic White and 51.4% non-Hispanic other/multiple workers [48]. In August 2020, the share of Asians who teleworked was three times higher than the share of Hispanic or Latino workers who teleworked [52].

Little is known about the underlying causes of racial and ethnic (hereafter racial) disparities in teleworking or about the mechanisms through which race and ethnicity (hereafter race) might affect teleworking opportunities. One possible explanation for racial disparity in teleworking is differences in education, particularly in college education. The education–race literature shows that although some improvements have been observed in the past two decades, there are still wide racial gaps in test scores, involvement in gifted programs, and college graduation rates [53,54,55]. For instance, using the 2013 through 2015 federal data, Libassi showed that Black and Hispanic college students were less likely than White students to earn a bachelor’s degree or to specialize in science, technology, engineering and math (STEM) fields [55]. Racial disparity in educational outcomes and professional achievements would affect the likelihood of teleworking, by influencing employment trajectories such as pay levels, titles, benefits, and positions.

Occupation is also strongly associated with likelihood of teleworking. The literature shows that certain racial groups are overrepresented and underrepresented in some occupations, relative to their share in the total workforce [56,57,58,59,60]. For instance, using the data from the Bureau of Labor Statistics Current Population Survey, Hawkins [56] showed that people of color were more likely to be employed in occupations with high potential exposure risk to infections. Asfaw [58] also showed that Black and Hispanic workers were overrepresented in occupations with high potential risk of exposure to disease and inability to work from home, respectively. Overrepresentation of some racial groups in specific occupations is expected to have a substantive influence on access to different benefits, including the opportunity to telework. Jobs in some occupations such as construction, agriculture, transportation, meat processing, and protective services cannot be performed remotely because it is practically impossible to perform the tasks at home. As indicated by Bui et al. [61], Hispanic and nonwhite workers are disproportionately employed in these occupations. This study brought together these two factors, education and occupation, to estimate the extent to which racial disparity in teleworking was mediated by these variables.

The objective of this study was to examine racial disparities in teleworking during the pandemic and to estimate the extent to which these disparities are explained through mediators like education and occupation and racial discrimination. Understanding these issues and quantifying the relative role of mediators and direct impacts of race in teleworking would help to target factors that perpetuate racial disparities in teleworking.

## 2. Materials and Methods

### 2.1. Data and Variables

The data source for this study was the Bureau of Labor Statistics (BLS) Current Population Survey (CPS). CPS is a nationally representative monthly survey that provides comprehensive data on labor force characteristics and other demographic information on the U.S. population. Every month, by means of a probability sampling method, about 60,000 occupied households are selected for interview. Selected respondents are interviewed for four consecutive months a year, for two years. This study considered respondents who were interviewed during May 2020 through July 2021 and were working during the survey weeks. This study did not consider respondents whose race was recorded as “other” and “mixed”; these categories accounted for 2.5% of the sample. CPS data organized by Integrated Public Use Microdata Series (IPUMS) were used [62].

Outcome variable: Starting in May 2020, BLS added five questions to the CPS to measure the effects of the COVID-19 pandemic on labor markets. One of the questions was “At any time in the last 4 weeks, did you telework or work at home for pay because of the coronavirus pandemic?” This question was used to generate a binary outcome variable (one if yes, and zero if no) that would measure teleworking because of the pandemic (hereafter referred to as teleworking).

Independent variable: “Race and ethnicity” was our independent variable. The CPS collects information on the race and ethnicity of respondents as White, Black, Asian, and any other race or race in combination. The survey also collects information on the ethnicity of respondents as Hispanic or non-Hispanic. This information was used in defining the race and ethnicity variable as non-Hispanic White (hereafter, White), non-Hispanic Black (hereafter, Black), non-Hispanic Asian (hereafter, Asian), and Hispanic. Workers whose ethnicity was identified as Hispanic could be of any race.

**Figure 1:**
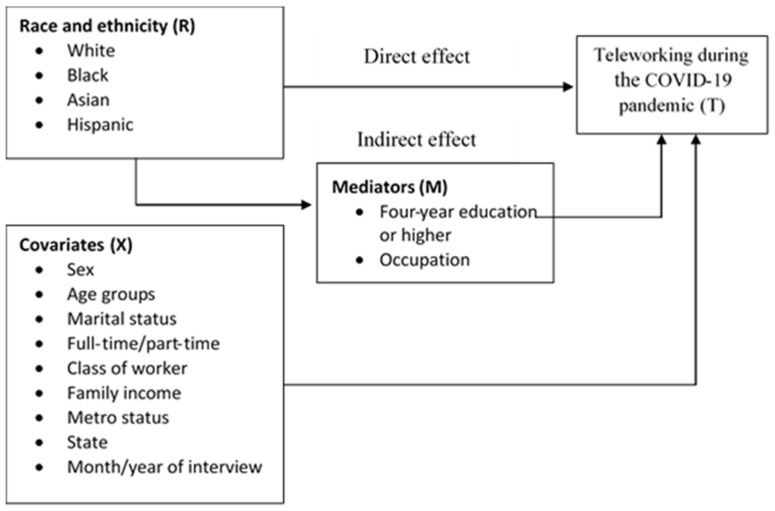
Association between racial disparities in teleworking during the COVID-19 pandemic: a mediation analysis.

Mediator variables: Variables that have strong association with both race and teleworking can be used as mediators. In this study, based on the literature presented in the introduction above, college education and occupation were identified as mediators of the relationship between race and teleworking (see Figure 1 below). In the survey, respondents were asked about the highest grade they completed. The study used this information to identify respondents with a four-year college degree or higher education level (hereafter referred to as four-year college education).

Respondents were also asked about their occupation. More than 400 occupations were reported in the CPS data. The BLS Standard Occupational Classification (SOC) system available at https://www.bls.gov/soc/2018/#classification (accessed on 10 July 2021) was used to aggregate the CPS occupations into 22 two-digit SOCs [63]. The BLS SOC classifies workers into different occupational categories. The 2018 US SOC has 867 eight-digit, 459 six-digit, 98 three-digit, and 23 two-digit groups.

Covariates: Some variables might affect the direct and indirect relationship of race on teleworking. To control for this effect, the following variables were included as covariates: sex; age (16–24, 25–34, 35–54, 55–64, 65+); marital status (married; widowed, divorced, or separated; never married); family income ($34,999 or less, $35,000–$74,999, $75,000–$149,999, $150,000 & above); class of worker (private, public, self-employed); full-time or part-time status; and geographical information including metropolitan or central city (central city, unknown, not in metro area, and outside central city) and state. Survey month/year (May 2020 through July 2021) was also included as a covariate to account for trends in teleworking, unemployment rate, and other time-variant macro variables. Industry was not included as a covariate because it is highly correlated with the occupation variable.

### 2.2. Mediation Analysis

As shown in Figure 1, controlling for covariates, race can influence the ability of workers to telework, either directly (that is, have a direct racial effect on teleworking) or indirectly, by affecting the mediator variables. The mediator variables are assumed to be a step in the pathways between race and teleworking. To test this assumption, the following two equations were estimated.

(1) $$T=\alpha_{0}+\beta X+\gamma R+\mu$$

(2) $$T={\alpha^{\prime }}_{0}+\beta^{\prime }X+\gamma^{\prime }R+\delta M+\varepsilon$$

where *T* is the outcome variable (teleworking due to the pandemic), *X* is a vector of covariate variables, *R* is the independent variable (race), *M* is a vector of mediator variables, *μ* and *ε* are the error terms of Equations (1) and (2), respectively. Parameters *α*_0_, *α*′_0_, *γ*, and *γ*′ and vector parameters *β*, *β*′, and *δ* are coefficients to be estimated from Equations (1) and (2).

Equation (1) is a reduced model (without mediator variables) and Equation (2) is the full model (with mediator variables). In Equation (1) *γ* measures the total effect of race on teleworking and in Equation (2) *γ*′ and *δ* measure the direct and indirect effects of race on teleworking, respectively. The vector parameter *δ* may partially or entirely explain the racial disparities in teleworking.

As indicated above, the outcome variable (T) was binary. Therefore, a logistic regression was estimated and a user-written Stata command, KHB (Karlson-Holm-Breen) was used, which decomposes the direct and indirect effects for nonlinear models [64]. The KHB method also allows use of more than mediators and decomposing of the indirect effect of each mediator. Most of the CPS respondents were interviewed more than once (66%), and this might violate the assumption of independence of observations. To address this problem, the Huber-White Sandwich estimator was used. In addition, survey weights were used to achieve national representativeness and to incorporate oversampling of specific groups and subgroups. Stata/SE 17.1 was used for all analyses.

## 3. Results

The final study sample comprised 702,500 observations. These observations represented 145 million White, Black, Asian, and Hispanic workers who worked during the study period. The shares of White, Black, Asian, and Hispanic workers in the total workforce were 63.8%, 11.7%, 6.4%, and 18.1%, respectively. Table 1 presents the descriptive statistics of the outcome, mediator, and covariate variables by race. This appendix shows that 22.3% of workers (95% confidence interval, or CI: 22.1% to 22.5%) teleworked during May 2020 through July 2021. There were statistically significant differences in the percentages of workers who teleworked, by race. Whereas 38.3% of Asian workers (95% CI: 37.5% to 39.2%) and 23.7% of White workers (95% CI: 23.5% to 24.0%) teleworked during the study period, 18.6% of Black workers (95% CI: 18.1% to 19.2%) and 13.9% of Hispanic workers (95% CI: 13.5% to 14.2%) teleworked. A two-tailed *t* test was used to test whether the percentage of Black, Asian, or Hispanic workers who teleworked was greater or less than the percentage of White workers who teleworked during the study period. The results showed that the differences were statistically significant (two-tailed *t*-test: White vs. Asian, *t* = −61.8, *p* < 0.01; White vs. Black, *t* = 22.5, *p* < 0.01; White vs. Hispanic, *t* = 61.3, *p* < 0.01).

Table 2 presents the regression results for the reduced (without mediator variables) and full model (with mediator variables). During May 2020 through July 2021, controlling for covariates, the odds for Black and Hispanic workers to telework were 35% (95% CI: 38% to 32%) and 55% (95% CI: 57% to 53%) lower, respectively, than for White workers, the reference category. However, the odds for Asian workers to telework were 44% higher (95% CI: 37% to 51%) than for White workers. All these associations were statistically significant and consistent with the descriptive results. After including the mediator variables, the odds of teleworking decreased for all racial groups.

Table 3 presents the total, direct, and indirect effects of race on the odds of teleworking, using the KHB mediation analysis method. The mediator variables, four-year college education and occupation, explained 83% and 78% of the disparities in the odds of teleworking for Black and Hispanic workers, respectively. For Asian workers, the mediators explained 64% of the disparities in the odds of teleworking. Because of the use of two mediators, the next important question to address was which of the mediators contributed most to the observed indirect effects of race on teleworking. The last two columns of Table 3 present the contribution of each mediator separately. For Black and Hispanic workers, having a four-year college education explained 25% and 28%, respectively, of the negative effect of race on the odds of teleworking. For Asian workers, having a four-year college education explained 19% of the total positive effect of race on the odds of teleworking. Occupation explained 64% and 63%, respectively, of the total negative effects of race on the odds of teleworking for Black and Hispanic workers. In addition, occupation explained 31% of the positive impact of race on the odds of teleworking for Asian workers.

Race still had a statistically significant association with teleworking (column 4 of Table 3). Being Black or Hispanic, in comparison with being White, decreased the odds of teleworking by 7% (95% CI: 11% to 3%) and 16% (95% CI: 19% to 12%), respectively, after controlling for covariates and mediators. Being Asian increased the odds of teleworking by 13% (95% CI: 8% to 20%) in comparison with being White.

**Figure 2:**
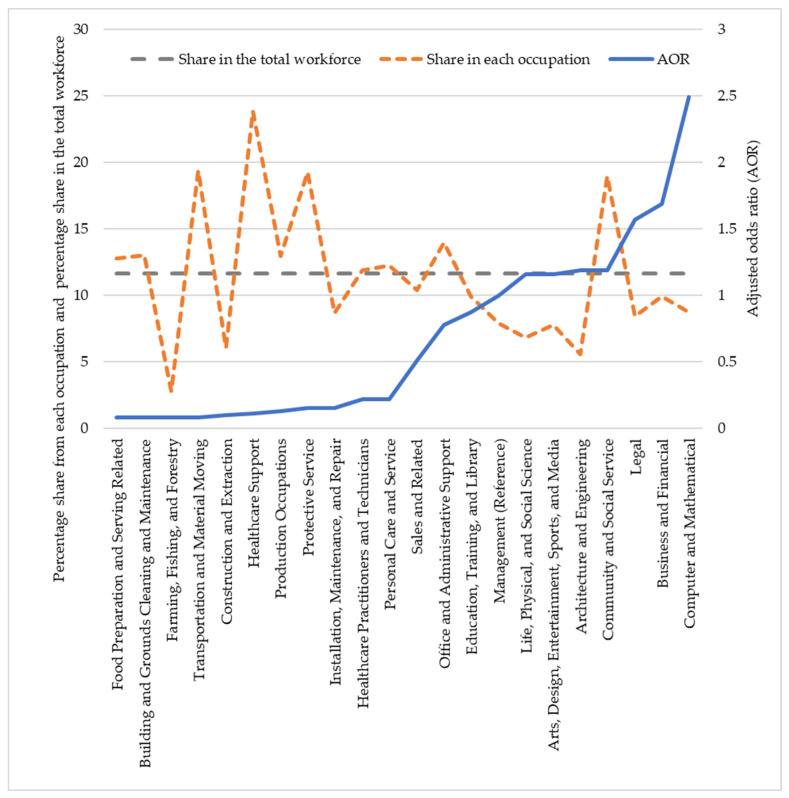
Adjusted odds ratio (AOR) of teleworking in each occupation and share of Black workers in each occupation and in the total workforce.

**Figure 3:**
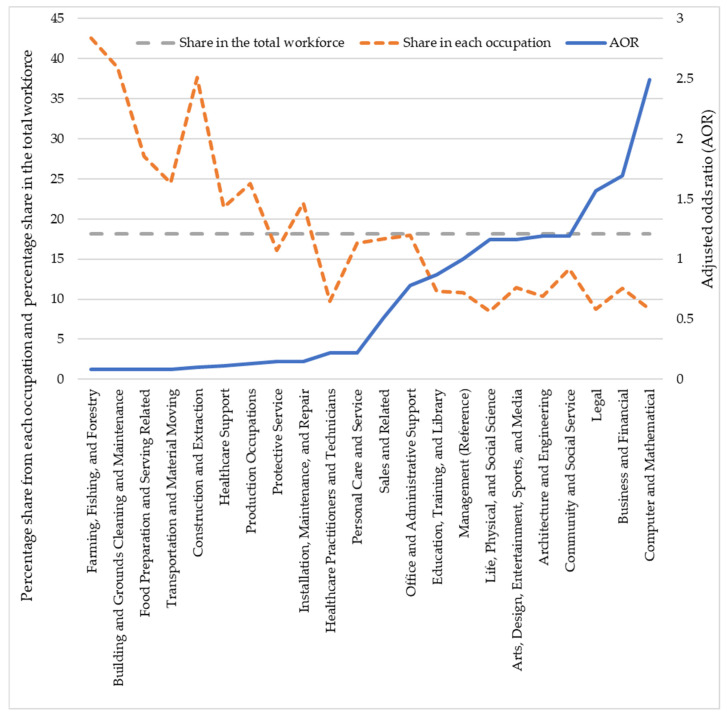
Adjusted odds ratio (AOR) of teleworking in each occupation and share of Hispanic workers in each occupation and in the total workforce.

**Figure 4:**
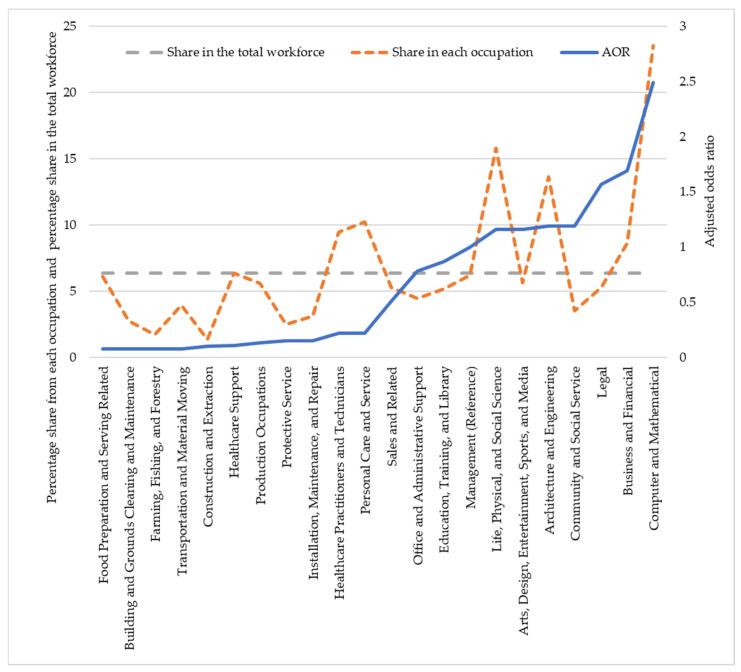
Adjusted odds ratio (AOR) of teleworking in each occupation and share of Asian workers in each occupation and in the total workforce.

Figure 2, Figure 3 and Figure 4 illustrate how race operates through occupation. These figures plotted the share of racial groups in different occupations and in the total workforce and the odds of teleworking in each occupation presented in Table 2.

**Figure 5:**
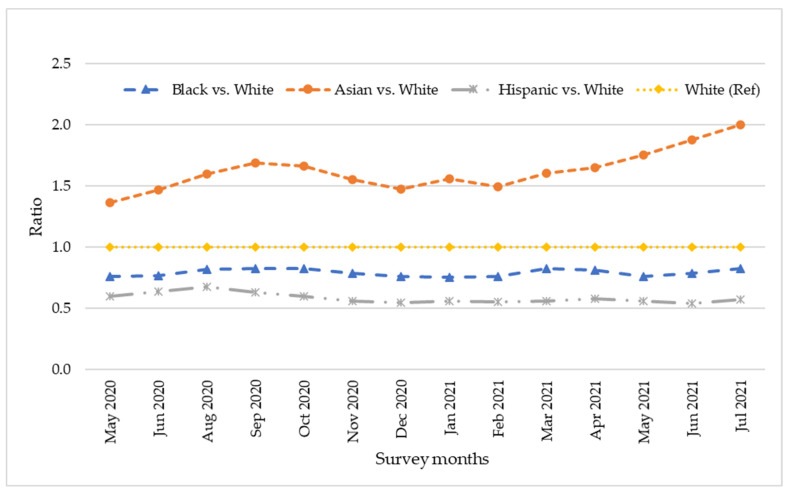
Ratio of Black, Asian, and Hispanic workers who teleworked relative to White workers who teleworked, by CPS survey months.

The study also examined whether there was any improvement in racial disparities in teleworking during the study period of May 2020 through July 2021. The results presented in Figure 5 show that there were no changes in the percentage of Black, Asian, and Hispanic workers who teleworked, relative to the percentage of White workers who did, during May 2020 through July 2021.

## 4. Discussion

Teleworking is one of the strategies followed by employers to reduce the spread of COVID-19. In addition to facilitating flexibility and improving work–family balance [65,66] and well-being [67], teleworking reduces the risk of exposure to COVID-19 and influenza-like illnesses and the risk of unemployment for those who have jobs that can be done remotely. However, despite an overall improvement in teleworking during the pandemic [68,69], marked racial disparities persist. During May 2020 through July 2021, the odds for Black and Hispanic workers to telework due to the pandemic were much lower than for both White and Asian workers after controlling for covariates.

Prior studies, however, have not examined the mechanisms through which race might affect disparities in teleworking. The hypothesis of this study was that racial disparities in teleworking could be mediated by differences in college educational attainment and occupation. The results showed that around 80% of the total variation in the odds of teleworking between White workers and Black and Hispanic workers was mediated by differences in four-year college education and occupation. This finding indicated that narrowing the gap in four-year college education and reducing disparities in the distribution of Black and Hispanic workers in different occupations relative to their share in the total workforce could substantially reduce the gap. For Asian workers, 64% of the total variation in the odds of teleworking was mediated by four-year college education and occupation, and the remaining 36% was unexplained. Future studies might explore other factors that would explain this gap.

The study also examined the contribution of each mediator to the total effect. A four-year college education mediated 25% and 28% of the indirect impact of race on teleworking for Black and Hispanic workers, respectively. This implies that, keeping all other factors constant, if the percentage of Black and Hispanic workers with a four-year college education was like that of White workers, disparities in the odds of teleworking between White and Black workers and between White and Hispanic workers could have been reduced by 25% and 28%, respectively. As shown in Table 1, the percentage of Black and Hispanic workers with a four-year college education was 27% and 51% lower, respectively, than the percentage of White workers.

Occupation explained more than 60% of the total effect of race on teleworking for Black and Hispanic workers. As shown in Figure 2, Black workers were overrepresented in occupations not suitable for teleworking, such as healthcare support, transportation and material moving, protective services, and building and grounds cleaning and maintenance occupations; the share of Black workers in these occupations was 104%, 66%, 65%, and 12% higher, respectively, than their share in the total workforce. The odds for workers to telework in these occupations were between 99% and 80% lower than for workers in the managerial (reference) occupations (Figure 2). However, in occupations with odds > 1 for teleworking, Black workers were underrepresented in all but one occupation (community and social assistance). These results showed that the impact of overrepresentation and underrepresentation of Black workers in different occupations relative to their share in the total workforce indirectly affected their ability to telework.

Similarly, in all occupations in which Hispanic workers were overrepresented relative to their share in the total workforce, the odds of teleworking were more than 85% lower than in the managerial occupations (Figure 3). For instance, the odds of teleworking in farming, fishing, and forestry and in building and grounds cleaning and maintenance occupations were 96% lower than in the management occupations; in these occupations, the shares of Hispanic workers were 134% and 115% higher than in the total workforce. Conversely, in all occupations in which the odds of teleworking were >1, the share of Hispanic workers was lower than their share in the total workforce. For instance, the odds of teleworking in computer and mathematical and legal occupations were 148% and 57% higher than in the management occupations; however, the shares of Hispanic workers in these two occupations were 52% lower than in the total workforce (Figure 3).

Similar results were reported by other researchers. For instance, Hawkins concluded that occupational segregation of people of color into high-risk industries and occupations increased their risk of exposure to COVID-19 [56]. Other studies also showed that Black workers were overrepresented in occupations such as occupational therapy and physical therapy assistants and aides; healthcare diagnosis or treatment practitioners; health technologists and technicians; other healthcare support; funeral service; and protective service, where the chances of teleworking were very slim [57,58,68]. Asfaw also showed that in occupations in which Hispanic workers were overrepresented relative to their share in the total workforce, the expected score for inability to work from home was 63% higher (95% CI: 1.32 to 1.99) than in occupations where they were underrepresented [58].

A different picture was observed in the case of Asian workers (Figure 4). In occupations in which the odds of teleworking were very small, the shares of Asian workers were also very low compared to their share in the total workforce, and vice versa. For instance, the shares of Asian workers in occupations for which teleworking was nearly impossible (AOR < 0.1)—farming, fishing, and forestry; building and grounds cleaning and maintenance; transportation and material moving; and construction and extraction occupations—were 73%, 56%, 38%, and 79% lower than their share in the total workforce. Contrarily, in computer and mathematical occupations, for which the odds of teleworking were 2.5 times higher than in the managerial occupations, the share of Asian workers was 270% higher than their share in the total workforce. Using the CPS data, Gaffney et al. [48] also showed that managers and software developers were among the five occupations with the most teleworkers. Mayo et al. [70] also showed that workers in high-tech and knowledge-based occupations were more likely to telework.

Finally, the statistically significant coefficients of the race variables, after controlling for covariates and mediator variables, might indicate the possibility of discrimination against Black and Hispanic workers in teleworking. The direct impact of race on the odds of teleworking was negative for Hispanic and Black workers (odds ratio < 1) and positive for Asian workers, and the coefficients were statistically significant (Table 3). After controlling for covariates and the effects of mediators, the odds for Hispanic and Black workers to telework were 16% and 7% lower than for White workers, respectively. The odds for Asian workers to telework were 13% higher than for White workers, after controlling for covariates and the effects of mediators. These results revealed that the possibility of racial discrimination in teleworking could not be ruled out. Employers or managers might be more reluctant to allow Black and Hispanic workers to telework than they were for White and Asian workers. Using the U.S. Census Household Pulse Survey, Ong and Ray [45] also reported statistically significant racial disparity in teleworking after controlling for income and education.

The results of this study also showed that there was no improvement in racial disparity in teleworking during the study period. As shown in Figure 5, the proportion of Black and Hispanic workers who teleworked relative to White workers remained the same during May 2020 through July 2021. For instance, during May 2020 through July 2021, the average monthly change in the percentage of Black and Hispanic workers who teleworked relative to that of White workers was <0.1%. This confirmed the results of the mediation analysis that the root problem of racial disparities in teleworking was more structural in nature, and there is no easy solution in the short run.

This study has some limitations. First, the CPS telework data did not include measurement of the intensity of telework. It was also not clear if this question was asked about the main or other job(s). However, the percentage of respondents with more than one job was less than 5% during the study period. Second, all the information in the CPS data was self-reported. Third, the CPS occupations were aggregated into 22 two-digit SOCs. This might camouflage disparities in teleworking and overrepresentation and underrepresentation of workers employed in some occupations such as meat packing and software engineering. The study also considered occupations at main jobs and not at other job(s). Fourth, education was measured in terms of 4-year or more college education and this excluded workers with an associate degree. However, the results were similar but less strong when education was measured in terms of associate degree and above [AOR 2.26; 95% CI 2.20–2.33]. Fifth, income can be considered as a mediator variable because it is highly correlated with race and teleworking. For instance, using a simple logit model, Ong and Ray [45] reported part of the racial disparities in teleworking operates through existing racial disparities in income and education. Future studies could consider income and other potential variables as additional mediator variables. Finally, mediation analysis implies causation. However, the study design and the cross-sectional nature of the data might not enable us to establish causal relationship between race and teleworking.

## 5. Conclusions

The COVID-19 pandemic demonstrated the importance of teleworking in protecting employees from health hazards and losing their jobs. However, structural problems related to race hampered the equal distribution of this opportunity across different racial groups. The current literature in this area clearly demonstrated racial disparities in teleworking. This study contributes to the literature by examining the mechanisms through which race affects disparities in teleworking during the COVID-19 pandemic. More than 80% of the disparities in teleworking between Black and Hispanic workers and White workers were related to the long-term impact of racial discrimination in college education and occupation. Disparities in four-year college education attainment explained more than one-quarter of the disparities in teleworking between Black and Hispanic workers and White workers. The results also showed overrepresentation and underrepresentation of Black and Hispanic workers in some occupations, relative to their share in the total workforce, contributed to more than 60% of the disparities in teleworking.

This study complements previous research findings that occupation plays an important role in the disproportionate impact of COVID-19 and other contagious diseases on some minority workers. Future research might explore why certain racial groups were overrepresented and underrepresented in some occupations. The study also found that race directly explained some of the disparities in teleworking, indicating the possibility of racial discrimination in teleworking. Finally, the trend analysis showed that there was no sign of improvement in racial disparities in teleworking in the previous 15 months. This finding indicates that there may be no short-term solutions to the problem. Ultimately, reducing racial disparities in college education and distribution of workers across different occupations would be a long-term solution for reducing racial disparities in teleworking.

## Figures and Tables

**Table 1:** Descriptive statistics: Current Population Survey (May 2020 through July 2021).

Variables	Total	Race and Ethnicity (Race)			
		White	Black	Asian	Hispanic
Number of observations	702,500	499,976	64,376	40,550	97,598
Number of weighted observations (thousands)	145,053	92,605	16,903	9270	26,276
Percentage (weighted)	100	63.8	11.7	6.4	18.1
**Outcome variable**					
Teleworking because of the pandemic (%)	22.3	23.7	18.6	38.3	13.9
**Mediator variables (%)**					
Four-year college degree	41.1	45.25	32.89	67.53	22.28
Occupation (row %)					
Management		75.0	7.9	6.2	10.9
Business and Financial		70.1	9.9	8.7	11.4
Computer and Mathematical		59.0	8.7	23.5	8.7
Architecture and Engineering		70.4	5.5	13.7	10.4
Life, Physical, and Social Science		68.9	6.8	15.8	8.5
Community and Social Service		63.8	19.0	3.5	13.7
Legal		77.5	8.4	5.3	8.7
Education, Training, and Library		73.9	9.9	5.2	11.0
Arts, Design, Entertainment, Sports, and Media		75.1	7.8	5.6	11.5
Healthcare Practitioners and Technicians		68.9	11.9	9.4	9.8
Healthcare Support		48.3	23.8	6.4	21.5
Protective Service		62.2	19.2	2.5	16.1
Food Preparation and Serving Related		53.3	12.8	6.1	27.9
Building and Grounds Cleaning and Maintenance		45.3	13.0	2.8	38.9
Personal Care and Service		60.5	12.3	10.2	17.0
Sales and Related		66.8	10.4	5.3	17.6
Office and Administrative Support		63.6	14.0	4.5	17.9
Farming, Fishing, and Forestry		53.0	2.8	1.7	42.5
Construction and Extraction		54.9	6.1	1.4	37.7
Installation, Maintenance, and Repair		66.2	8.7	3.1	22.0
Production Occupations		57.0	13.0	5.6	24.4
Transportation and Material Moving		52.1	19.4	4.0	24.5
**Control variables**					
Male (%)	53.2	53.1	47.1	53.3	57.2
Age category (row %)					
16–24		60.1	12.0	4.2	23.7
25–34		59.1	13.1	7.3	20.5
35–54		61.8	11.9	7.3	19.1
55–64		71.9	10.3	5.3	12.5
65+		77.7	8.7	4.6	9.0
Marital status (column %)					
Married		67.9	7.9	7.7	16.6
Widowed, divorced, or separated		66.7	13.7	3.1	16.5
Never married		55.9	17.2	5.5	21.4
Family income					
$34,999 or less		47.6	18.8	4.4	29.1
$35,000–$74,999		58.8	14.1	4.7	22.5
$75,000–$149,999		69.7	9.5	6.1	14.8
$150,000 or more (Reference)		72.9	6.8	10.7	9.6
Full-time status (row %)					
Full-time		65.4	10.8	5.6	18.2
Part-time		63.4	11.9	6.6	18.1
Class of worker (row %)					
Public		7.5	5.3	15.6	71.7
Private		11.6	6.8	19.3	62.3
Self-employed		15.2	5.0	13.4	66.5

White = non-Hispanic White; Black = non-Hispanic Black; Asian = non-Hispanic Asian.

**Table 2:** Association between teleworking and race.

	Without Controlling for the Effects of Mediators			Controlling for the Effects of Mediators		
	AOR	95% CI		AOR	95% CI	
Independent variable						
Race						
White (Reference)						
Black	0.65	0.62	0.68	0.93	0.89	0.97
Asian	1.44	1.37	1.51	1.14	1.08	1.20
Hispanic	0.45	0.43	0.47	0.84	0.81	0.88
Mediator variables						
Four-year degree or higher				2.43	2.36	2.50
Occupation						
Management (Reference)						
Business and Financial				1.69	1.62	1.77
Computer and Mathematical				2.49	2.35	2.63
Architecture and Engineering				1.19	1.11	1.28
Life, Physical, and Social Science				1.16	1.06	1.28
Community and Social Service				1.19	1.11	1.28
Legal				1.57	1.45	1.70
Education, Training, and Library				0.87	0.83	0.92
Arts, Design, Entertainment, Sports, and Media				1.16	1.09	1.25
Healthcare Practitioners and Technicians				0.22	0.21	0.23
Healthcare Support				0.11	0.10	0.12
Protective Service				0.15	0.13	0.16
Food Preparation and Serving Related				0.08	0.07	0.09
Building and Grounds Cleaning and Maintenance				0.08	0.07	0.09
Personal Care and Service				0.22	0.19	0.24
Sales and Related				0.51	0.49	0.53
Office and Administrative Support				0.78	0.75	0.82
Farming, Fishing, and Forestry				0.08	0.06	0.10
Construction and Extraction				0.10	0.09	0.11
Installation, Maintenance, and Repair				0.15	0.14	0.17
Production Occupations				0.13	0.12	0.15
Transportation and Material Moving				0.08	0.07	0.08
Covariate						
Male	0.57	0.55	0.58	0.84	0.82	0.86
Age category						
16–24 (Reference)	1.00	1.00	1.00	1.00	1.00	1.00
25–34	3.18	3.01	3.36	1.82	1.72	1.92
35–54	2.77	2.62	2.93	1.73	1.64	1.83
55–64	2.27	2.14	2.42	1.56	1.46	1.65
65+	2.42	2.26	2.60	1.37	1.28	1.47
Marital status	1.00	1.00	1.00	1.00	1.00	1.00
Married (Reference)						
Widowed, divorced, or separated	1.00	0.96	1.04	1.08	1.04	1.12
Never married	1.01	0.98	1.04	1.05	1.01	1.08
Family income						
$34,999 or less	0.19	0.18	0.20	0.57	0.54	0.60
$35,000–$74,999	0.26	0.25	0.27	0.61	0.59	0.63
$75,000–$149,999	0.49	0.48	0.51	0.76	0.74	0.79
$150,000 & above (Ref)						
Full-time status						
Part-time worker (Reference)						
Full-time worker	1.38	1.35	1.42	1.08	1.05	1.11
Class of worker						
Public (Reference)						
Self-employed	0.38	0.36	0.40	0.47	0.45	0.50
Private	0.50	0.48	0.52	0.76	0.73	0.78
Metro status						
Central city (Reference)						
Unknown	0.35	0.33	0.37	0.50	0.48	0.53
Not in metro area	0.19	0.18	0.20	0.33	0.31	0.35
Outside central city	0.62	0.61	0.64	0.75	0.72	0.77
States (51 state dummies)						
Period (15 month/year dummies)						
Number of observations		702,500				

AOR = adjusted odds ratio; CI = confidence interval.

**Table 3:** Total, direct, and indirect effects of race on teleworking: CPS May 2020 through July 2021.

Race	Odds Ratio (95% Confidence Interval)			Variation Explained by Mediators (%)		
	Total Effect ^†^	Indirect Effect ^§^	Direct Effect ^‡^	Total	College Degree	Occupation
White (Reference)						
Black	0.65 (0.62–0.68)	0.70 (0.67–0.73)	0.93 (0.89–0.97)	83.1	25.4	63.7
Asian	1.44 (1.37–1.51)	1.26 (1.21–1.32)	1.13 (1.08–1.20)	64.1	18.6	31.1
Hispanic	0.45 (0.43–0.47)	0.54 (0.51–0.56)	0.84 (0.81–0.88)	78.1	28.4	62.8

^†^ Reduced model, adjusting for covariates. ^§^ The indirect effect is the difference between the total and the direct effects, and it measures the effect of race through mediators. ^‡^ Full model.

## Data Availability

The data that support the findings of this study are available https://cps.ipums.org/cps/ upon request (accessed on 11 August 2021).
